# A urine-based DNA methylation assay, ProCUrE, to identify clinically significant prostate cancer

**DOI:** 10.1186/s13148-018-0575-z

**Published:** 2018-11-23

**Authors:** Fang Zhao, Ekaterina Olkhov-Mitsel, Shivani Kamdar, Renu Jeyapala, Julia Garcia, Rachel Hurst, Marcelino Yazbek Hanna, Robert Mills, Alexandra V. Tuzova, Eve O’Reilly, Sarah Kelly, Colin Cooper, The Movember Urine Biomarker Consortium, Colin Cooper, Bharati Bapat, Rob Bristow, Chris Parker, Ian Mills, Hardev Pandha, Hayley Whitaker, David Neal, Mireia Olivan, Hing Leung, Antoinette Perry, Martin Sanda, Jack Schalken, Daniel Brewer, Antoinette S. Perry, Jeremy Clark, Neil Fleshner, Bharati Bapat

**Affiliations:** 1 Lunenfeld-Tanenbaum Research Institute, Sinai Health System, Toronto, Canada; 2 Department of Laboratory Medicine & Pathobiology, University of Toronto, Toronto, Canada; 3 Schools of Medicine and Biological Sciences, University of East Anglia, Norwich, Norfolk UK; 4 Norfolk and Norwich University Hospital, Norwich, Norfolk UK; 5 Cancer Biology and Therapeutics Laboratory, School of Biomolecular and Biomedical Science, Conway Institute, University College Dublin, Dublin 4, Ireland; 6 The Earlham Institute, Norwich, Norfolk UK; 7 Division of Urology, University Health Network, University of Toronto, Toronto, Canada

**Keywords:** Prostate cancer, PSA, Urine, DNA methylation, Biomarker, Early detection, Overtreatment

## Abstract

**Background:**

Prevention of unnecessary biopsies and overtreatment of indolent disease remains a challenge in the management of prostate cancer. Novel non-invasive tests that can identify clinically significant (intermediate-risk and high-risk) diseases are needed to improve risk stratification and monitoring of prostate cancer patients. Here, we investigated a panel of six DNA methylation biomarkers in urine samples collected post-digital rectal exam from patients undergoing prostate biopsy, for their utility to guide decision making for diagnostic biopsy and early detection of aggressive prostate cancer.

**Results:**

We recruited 408 patients in risk categories ranging from benign to low-, intermediate-, and high-risk prostate cancer from three international cohorts. Patients were separated into 2/3 training and 1/3 validation cohorts. Methylation biomarkers were analyzed in post-digital rectal exam urinary sediment DNA by quantitative MethyLight assay and investigated for their association with any or aggressive prostate cancers.

We developed a Prostate Cancer Urinary Epigenetic (ProCUrE) assay based on an optimal two-gene (*HOXD3* and *GSTP1*) LASSO model, derived from methylation values in the training cohort, and assessed ProCUrE’s diagnostic and prognostic ability for prostate cancer in both the training and validation cohorts.

ProCUrE demonstrated improved prostate cancer diagnosis and identification of patients with clinically significant disease in both the training and validation cohorts. Using three different risk stratification criteria (Gleason score, D’Amico criteria, and CAPRA score), we found that the positive predictive value for ProCUrE was higher (59.4–78%) than prostate specific antigen (PSA) (38.2–72.1%) for all risk category comparisons. ProCUrE also demonstrated additive value to PSA in identifying GS ≥ 7 PCa compared to PSA alone (DeLong’s test *p =* 0.039), as well as additive value to the PCPT risk calculator for identifying any PCa and GS ≥ 7 PCa (DeLong’s test *p* = 0.011 and 0.022, respectively).

**Conclusions:**

ProCUrE is a promising non-invasive urinary methylation assay for the early detection and prognostication of prostate cancer. ProCUrE has the potential to supplement PSA testing to identify patients with clinically significant prostate cancer.

**Electronic supplementary material:**

The online version of this article (10.1186/s13148-018-0575-z) contains supplementary material, which is available to authorized users.

## Introduction

The introduction of circulating prostate specific antigen (PSA) test has increased the rate of diagnosis of prostate cancer (PCa) by as much as 50%. However, the majority of PCa patients diagnosed through PSA screening present with low-risk, localized, Gleason score (GS) 6 tumors. Although PSA has a high negative predictive value (NPV) for PCa, its lack of specificity, limited impact on reducing morbidity, and the harms of over-diagnosing indolent disease have raised concerns about PSA screening [1].

To reduce overtreatment and associated morbidity, the U.S. Preventive Services Task Force (USPSTF) recently recommended against PSA screening to prevent unnecessary biopsies of “clinically insignificant” PCa (CI-PCa) which included patients with benign and low-risk disease [1]. However, following these recommendations, there was a substantial decrease (42.9%) [2] in the detection of GS ≥ 7 disease, indicating the reduction in PSA screening could delay diagnosis of “clinically significant” PCa (CS-PCa) consisting of intermediate- and high-risk disease. The revised recommendations now include advising men under 70 about the potential benefits and limitations of PSA based screening. However, their impact on the diagnosis of CS-PCa is currently unknown.

Several nomograms have been developed to estimate PCa aggressiveness following biopsy, such as the well-established D’Amico criteria [3] which includes PSA, GS, and clinical T stage. Due to the limited number of variables, patients with the same D’Amico risk category may have vastly different outcomes. Alternatively, the recently developed UCSF-Cancer of the Prostate Risk Assessment (CAPRA) score [4] is more informative due to ease of calculation and inclusion of key clinical variables including age, PSA, percent of cores positive in biopsy (%core), clinical T stage, and Gleason patterns. There is also the Prostate Cancer Prevention Trial (PCPT) PCa risk calculator [5], which takes into account ethnicity, family history, PSA, age, and digital rectal exams (DREs) results to calculate the risk of finding any cancer or high-risk (GS ≥ 7) cancer upon biopsy. These nomograms are used to distinguish low-risk versus high-risk PCa patients for management decisions after biopsy.

Low-risk PCa patients may be recommended enrollment into an active surveillance (AS) protocol where they are monitored with DREs, PSA tests, multiparametric (mp) MRI where available, and periodic biopsies instead of definitive treatment [6]. Although AS is a preferable management option for patients with CI-PCa, many AS patients with indolent tumors still undergo additional unnecessary biopsies and suffer associated morbidities.

Consequently, there is an urgent need to develop non-invasive biomarkers to complement PSA screening for the early identification of aggressive PCa and to guide decision making for initial diagnostic prostate biopsy or repeat biopsies of low-risk patients on AS. To address this, the Movember foundation introduced the Global Action Plan (GAP) 1: Urine biomarker initiative, which brought together 12 research teams from seven different countries. Our study, as part of this initiative, investigated non-invasive DNA methylation biomarkers for improved prognostication of PCa.

Aberrant DNA methylation is a hallmark of PCa [7, 8]. Tumor-specific gene methylation alterations are ideal biomarkers due to their stability and ease of detection from patient samples with limited amounts of DNA such as urinary sediments. Detection of DNA methylation biomarkers in urine sediment is non-invasive and may be able to supplement PSA screening to identify CS-PCa patients.

We have previously discovered and/or characterized tumor-specific DNA methylation of six genes (*APC*, *GSTP1*, *HOXD3*, *KLK10*, *TBX15*, and *TGFβ2*) in radical prostatectomy tumor samples [9–11]. Increased methylation of these genes was found to be associated with higher GS and adverse clinical prognosis. We also examined these biomarkers in post-DRE urine samples from a Canadian AS PCa patient cohort [12]. In the current study, we investigated the utility of these urinary DNA methylation biomarkers for diagnosis and prognostication of CS-PCa in three international patient cohorts.

## Results

### Cohort characteristics

The clinicopathologic characteristics for patient cohorts are summarized in Table 1. To mitigate any inherent biases in patient recruitment, all patients were combined, randomized, and separated into training (2/3 of patients) and validation (1/3 of patients) cohorts [13, 14] (Table 2).

**Table 1:** Clinical characteristics from the University of East Anglia, UK (UEA), GU Biobank at UHN, Canada (UHN), Trinity College at Dublin, Ireland (Dublin)

Patient clinical characteristics	UEA	UHN	Dublin
*n* (%)	194 (48)	155 (38)	59 (14)
Benign	109 (56)	46 (30)	27 (46)
PCa	85 (44)	109 (70)	32 (54)
Gleason score			
6	17 (20)	64 (59)	15 (47)
7	42 (49)	32 (29)	9 (28)
8–10	26 (31)	13 (12)	8 (25)
Clinical T stage			
T1	38 (45)	91 (83)	20 (63)
T2	14 (16)	16 (15)	11 (34)
T3	19 (22)	2 (2)	1 (3)
T4	14 (16)	0	0
% Biopsy cores positive for PCa			
Median	57%	20%	21%
Range	7–100%	5–100%	6–100%
Interquartile range	33%–100%	9%–38%	13%–43%
N/A	9	1	0
Age at enrollment			
Median	67	64	65
Range	42–85	37–83	46–80
Interquartile range	62–73	57–69	58–71
PSA at presentation			
Median	8.4	5.8	5.9
Range	0.2–277.3	0.01–67.31	0.5–248
Interquartile range	5.8–12.2	3.97–9.07	3.85–8.64
Prostate volume			
Median	59.54	47	
Range	21.08–244.6	16.05–127.0	
Interquartile range	42.52–86.52	34–57	
N/A	92	18	59
Perineural invasion			
Yes	28	11	4
No	80	17	55
N/A	86	127	0
CAPRA risk			
CAPRA low	10 (12)	57 (52)	13 (41)
CAPRA intermediate	32 (38)	36 (33)	14 (44)
CAPRA high	43 (51)	16 (15)	5 (16)
D’Amico risk			
D’Amico low	8 (9)	52 (48)	9 (28)
D’Amico intermediate	29 (34)	39 (36)	13 (41)
D’Amico high	48 (56)	18 (17)	10 (31)

**Table 2:** Cohort characteristics of the training and validation cohorts

Patient clinical characteristics	Training	Validation
*n* (%)	268 (65.4)	140 (34.6)
Benign	123 (44)	59 (41)
PCa	145 (52)	81 (57)
Gleason score		
6	60 (41)	36 (44)
7	55 (38)	28 (35)
8–10	30 (21)	17 (21)
Clinical T stage		
T1	101 (70)	48 (59)
T2	25 (17)	16 (20)
T3	11 (8)	11 (14)
T4	8 (6)	6 (7)
% Biopsy cores positive for PCa		
Median	29%	33%
Range	5–100%	5–100%
Interquartile range	13%–55%	14%–63%
N/A	8	6
Age at enrollment		
Median	66	66
Range	42–85	37–85
Interquartile range	59–71	59–72
PSA at presentation		
Median	6.9	7.04
Range	0.01–248	0.04–377.00
Interquartile range	4.55–10.36	4.79–11
Prostate volume		
Median	7	49
Range	18.0–121.6	18.0–121.6
Interquartile range	39.15–68.68	37–65.1
N/A	114	58
Perineural invasion		
Yes	30	13
No	97	55
N/A	153	75
CAPRA risk		
CAPRA low	47 (32)	33 (41)
CAPRA intermediate	56 (39)	26 (32)
CAPRA high	42 (29)	22 (27)
D’Amico risk		
D’Amico low	41 (28)	28 (35)
D’Amico intermediate	57 (39)	24 (30)
D’Amico high	47 (32)	29 (36)

Age and PSA were significantly correlated with each other, as well as prostate volume, and %core. (Additional file 1: Table S1, Spearman’s ρ *p* < 0.01).

### Detection of urinary DNA methylation biomarkers and association with clinicopathologic variables

**Fig. 1:**
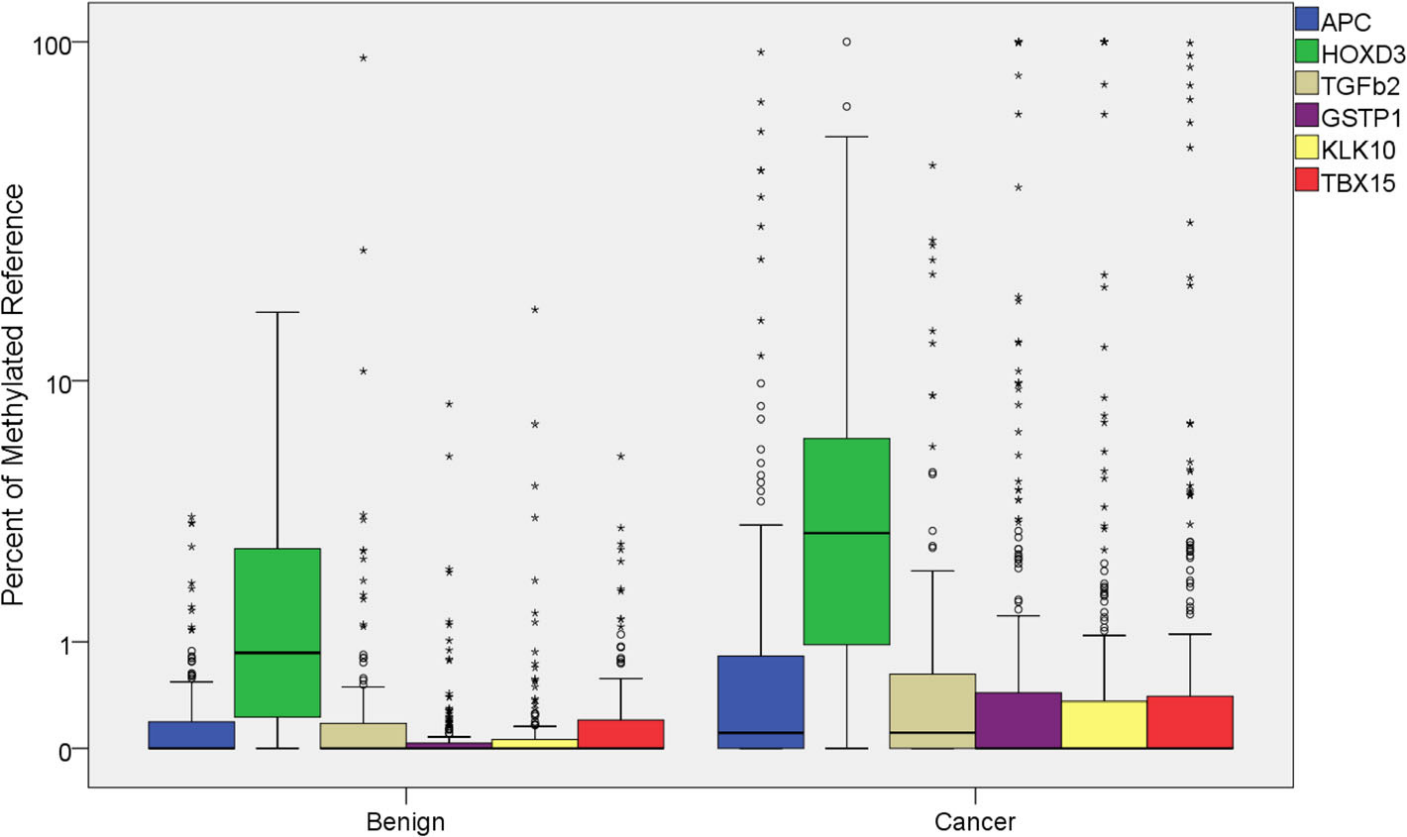
Distribution of percent of methylated reference (PMR) values for individual biomarkers among benign and PCa (Cancer) patients. Number of patients = 408. *APC*, *HOXD3*, *TGFβ2*, *GSTP1*, and *KLK10* are able to significantly differentiate benign and PCa (Mann Whitney *U p* < 0.05). Circles indicate outliers within 1.5× IQR, stars indicate outliers > 1.5× IQR. Mann Whitney *U p* values can be found in Additional file 1: Table S2

We assessed DNA methylation of our panel of biomarkers in the urinary sediment of patients recruited. Methylation frequencies (patients with percent methylated of reference (PMR) > 0) ranged from 39.5% (161/408 patients) for *GSTP1* to 92.6% (378/408 patients) for *HOXD3*. PMR distribution for individual markers among benign and PCa patients is shown in Fig. 1. Five of the six gene methylation showed significant increase in PCa compared to benign (Additional file 1: Table S2 Mann Whitney *U p* < 0.05).

Methylation levels of all six genes were significantly correlated with each other (Spearman’s ρ *p* < 0.01). *APC, GSTP1*, *KLK10*, *TBX15*, and *TGFB2* showed significant association with age and %core (Additional file 1: Table S1; Spearman’s ρ *p* < 0.05). Additionally, *GSTP1*, *KLK10*, and *TBX15* were associated with PSA (Spearman’s ρ *p* < 0.05). *HOXD3* did not correlate with any clinical variables.

### Building an optimal predictor gene model and ProCUrE assay

**Fig. 2:**
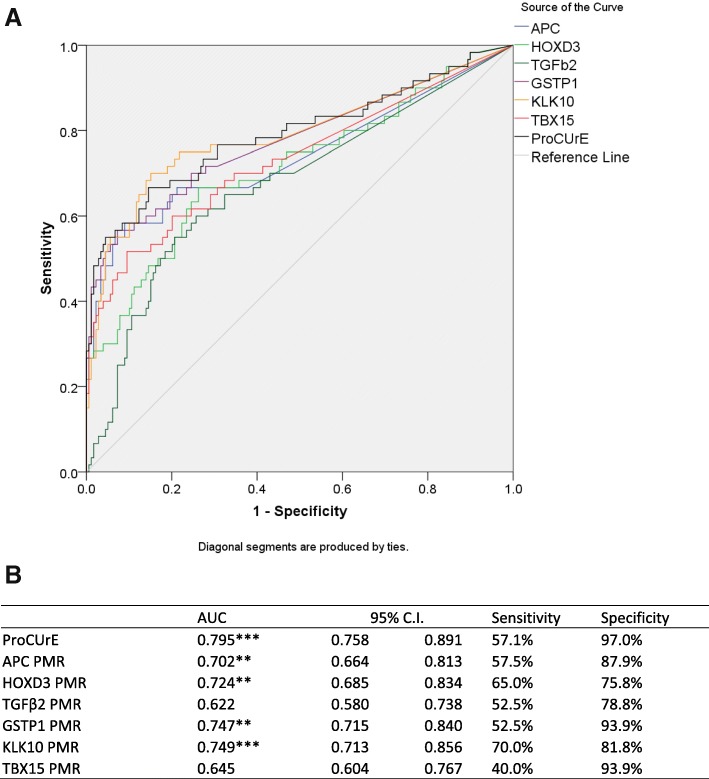
**a** Receiver operating characteristic (ROC) curves for individual biomarkers and ProCUrE, stratifying between benign (*n* = 123) and CAPRA high-risk (*n* = 42) patients in the training cohort. PSA was not included in this figure since PSA is used to calculate CAPRA and will always have a very strong association with CAPRA high risk. **b** AUC (bootstrapped 1000 iterations), sensitivity, and specificity for each gene and ProCUrE. ROC **p < 0.01; ***p < 0.001

To investigate whether combinations of biomarkers were more informative compared to individual markers for detection of any PCa and/or aggressive PCa, we applied least absolute shrinkage and selection operator (LASSO) and constructed an optimal two-gene (*HOXD3* and *GSTP1*) classifier model (ProCUrE) in the training cohort comparing between benign vs CAPRA-HR patients. Receiver operating characteristic (ROC) curve analysis of ProCUrE showed an area under curve (AUC) of 0.795 (bootstrapped 1000 iterations) (Fig. 2), which was higher than any individual marker; thus, we did not analyze individual markers in the validation cohort**.** An optimal cut-off threshold for ProCUrE was established with the maximum combined sensitivity (57.1%) and specificity (97%). Patients with methylation levels above this threshold are considered positive for ProCUrE status (ProCUrE +ve).

### Assessment of ProCUrE for improved PCa diagnosis

**Fig. 3:**
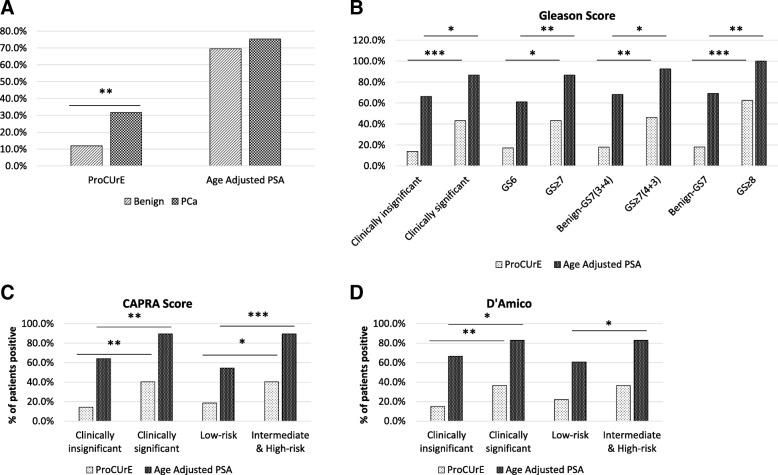
Diagnostic and prognostic ability of ProCUrE and age-adjusted PSA in the validation cohort. **a** The percent false- and true-positive for ProCUrE or age-adjusted PSA separating benign and PCa patients. **b** The percent of patients positive for ProCUrE or age-adjusted PSA for clinically insignificant (benign and low-risk) vs clinically significant (intermediate- and high-risk) based on Gleason score. **c**, **d** The percent of patients positive for ProCUrE or age-adjusted PSA for clinically insignificant (benign and low-risk) vs clinically significant (intermediate- and high-risk) and low-risk vs intermediate- and high-risk as determined by CAPRA score and D’Amico. *N* = 140, *χ*^2^*p < 0.05, **p < 0.01, ***p < 0.001

To determine ProCUrE’s value for PCa diagnosis, we tested its association with PCa. ProCUrE +ve status was significantly associated with PCa positive biopsies in both the training (Additional file 2: Figure S1A) and validation cohorts (Fig. 3a) (*χ*^2^ *p* < 0.01) while age-adjusted PSA (see definition in the “Material and methods” section) [15, 16] was not (Fig. 3a; *χ*^2^ *p* > 0.05). ProCUrE status identified 31.6% PCa patients with 11.9% false positive cases, while age-adjusted PSA detected 75.3% PCa patients but also had a high number (69.5%) of false positives. The positive predictive value (PPV) for ProCUrE was higher than for age-adjusted PSA (78.1% vs 59.8%) (Table 3A). These results demonstrate that ProCUrE +ve patients are more likely to harbor PCa.

**Table 3:** Diagnosis (A) and prognostication (B–D) of PCa

	PPV	NPV
A		
Benign vs PCa		
ProCUrE	78.10%	49.10%
Age-adjusted PSA	59.80%	47.40%
B		
GS clinically insignificant vs clinically significant		
ProCUrE	59.40%	76.40%
Age-adjusted PSA	38.20%	84.20%
GS6 vs GS ≥ 7		
ProCUrE	76.00%	53.70%
Age-adjusted PSA	63.90%	70.00%
Benign, GS6, GS7(3 + 4) vs GS ≥ 7 (4 + 3)		
ProCUrE	37.5%	86.8%
Age-adjusted PSA	24.5%	94.7%
Benign, GS6, GS7 vs GS ≥ 8		
ProCUrE	31.3%	94.3%
Age-adjusted PSA	16.7%	100.0%
C		
CAPRA clinically insignificant vs clinically significant		
ProCUrE	59.40%	73.60%
Age-adjusted PSA	42.20%	86.80%
CAPRA low risk vs intermediate and high risk		
ProCUrE	76.00%	48.10%
Age-adjusted PSA	70.50%	75.00%
D		
D’Amico clinically insignificant vs clinically significant		
ProCUrE	59.40%	68.90%
Age-adjusted PSA	43.10%	76.30%
D’Amico low risk vs intermediate and high risk		
ProCUrE	76.00%	38.90%
Age-adjusted PSA	72.10%	55.00%

Positive (PPV) and negative (NPV) predictive values for ProCUrE and age-adjusted PSA in the validation cohort separating benign vs PCa (A); clinically insignificant (benign and low-risk) vs clinically significant (intermediate- and high-risk) and low-risk vs clinically significant (intermediate- and high-risk) as determined by GS, CAPRA score, D’Amico criteria. (*χ*^2^ *p* values for these comparisons could be found in Fig. 3)

### Assessment of ProCUrE for early prognostication of PCa

To investigate ProCUrE’s value for PCa prognostication, we assessed the ability of individual markers, ProCUrE, and clinical variables to differentiate CI-PCa and CS-PCa patients as determined by GS. Using univariable logistic regression analysis, ProCUrE, PSA, and age showed significant association with CS-PCa. Due to the difference in range of each variable, interquartile range odds ratios (IQR OR) were estimated. The IQR OR of ProCUrE in the validation cohort (OR = 1.58, 95% CI = 1.28–1.96) were of similar size to PSA (OR = 1.98, 95% CI = 1.46–2.68), and age (OR = 1.66, 95% CI = 1.13–2.45) for CS-PCa (Table 4A). Multivariable logistic regression of significant variables age, PSA, and ProCUrE showed that ProCUrE was an independently significant variable for CS-PCa in the validation cohort (Table 4B). These results show that ProCUrE is a robust prognosticator of CS-PCa.

**Table 4:** Prediction of CS-PCa (as determined by GS) by individual markers, clinical variables, and ProCUrE

A							
Univariable	1st quartile	3rd quartile	Difference	OR	95% CI.		*p* value
ProCUrE	− 2.0006	− 1.0828	0.91789	1.58***	1.28	1.96	< 0.0001
APC	0	0.37652	0.37652	1.24***	1.10	1.39	0.0003
HOXD3	0.43965	5.2043	4.7646	1.54***	1.24	1.90	< 0.0001
GSTP1	0	0.1216	0.1216	1.06**	1.02	1.10	0.0013
KLK10	0	0.12665	0.12665	1.07**	1.02	1.11	0.0048
TGFβ2	0	0.309	0.309	1.03*	1.00	1.06	0.0481
TBX15	0	0.21295	0.21295	1.16***	1.08	1.24	< 0.0001
PSA	4.565	10.48	5.915	1.98***	1.46	2.68	< 0.0001
Age	59	71	12	1.66**	1.13	2.45	0.0105
PSA density	0.09	0.2	0.11	2.35***	1.51	3.68	0.0002
Prostate volume	40	70	30	0.70	0.47	1.06	0.0926
B							
Multivariable	OR	95% CI.			*p* value		
ProCUrE	1.358*	1.051	1.754		0.0194		
PSA	0.816	0.373	1.785		0.6108		
Age	2.718**	1.295	5.707		0.0082		
PSA density	2.878**	1.455	5.694		0.0024		

Using univariable and multivariable logistic regression, the ability of individual methylation markers, ProCUrE, and clinical variables to differentiate CI-PCa and CS-PCa as determined by GS was assessed in the training cohort. Since the scale of each variable is different, interquartile range odds ratios were estimated (logistic regression model *p < 0.05, **p < 0.01, ***p < 0.001)

We examined ProCUrE among patients stratified into different risk categories based on GS, D’Amico criteria and CAPRA score. *χ*^2^ analysis showed that both ProCUrE and age-adjusted PSA could differentiate between patients harboring no disease and/or CI-PCa versus CS-PCa in both the training cohort (Additional file 2: Figure S1B–D) and validation cohort (Fig. 3b–d) (*χ*^2^ *p* < 0.05). ProCUrE was able to differentiate low-risk vs intermediate- and high-risk PCa patients based on GS and CAPRA score, but not D’Amico criteria (Fig. 3b–d). Furthermore, ProCUrE’s prognostic value was consistently more robust as demonstrated by a more stringent *p* value (*χ*^2^ *p* < 0.01) compared to age-adjusted PSA (*χ*^2^ *p* < 0.05) in the validation cohort.

ProCUrE exhibited higher PPV than age-adjusted PSA for several different prognostic assessments (PPV: 76% vs 70.5% for CAPRA, 76% vs 72.1% for D’Amico, and 76% vs 63.9% for GS risk, Table 3B–D) indicating its overall robust ability to identify CS-PCa. Additionally, we found that ProCUrE significantly differentiated high-grade PCa, including GS ≥ 7(4 + 3), and GS ≥ 8 from all other patients, with higher PPV (37.5% and 31.3%, respectively) compared to age-adjusted PSA (24.5% and 16.7%, respectively) (Fig. 3, Table 3B–D).

These results indicate that patients who are ProCUrE +ve have a higher likelihood of harboring CS-PCa and high-grade (GS ≥ 8) tumors.

### Additional discriminative value of ProCUrE to PSA

To determine whether ProCUrE could add discriminatory value to PSA testing, we performed concordance statistics (c-statistic) analysis of PSA alone and ProCUrE with PSA combined in the training cohort using logistic regression. The c-statistic with PSA and ProCUrE combined (0.775) is significantly improved over PSA alone (0.729) (DeLong test *p* = 0.039), indicating that ProCUrE has additional discriminatory value to PSA for detecting CS-PCa (Table 5). Only GS risk was analyzed since CAPRA score and D’Amico criteria is calculated using PSA.

**Table 5:** C-statistic for distinguishing clinically significant disease based on GS

CI-PCa vs CS-PCa (GS)	C-statistic
PSA	0.729
ProCUrE	0.684
Combined	0.775*

C-statistics was used to determine any additive value of ProCUrE to PSA for discriminating CI-PCa vs CS-PCa as determined by GS in the training cohort. Only GS risk was analyzed since CAPRA score and D’Amico criteria is calculated using PSA. DeLong’s test *p = 0.039

### Additional discriminative value of ProCUrE to PCPT

To determine whether ProCUrE could add additional value to current clinical nomograms, we used the PCPT risk calculator for risk assessment in a subset of 144 patients (out of 408 patients in total) that had family history, ethnicity, and DRE results available.

We assessed the diagnostic (detection of any PCa) and prognostic (detection of GS ≥ 7 PCa) value of PCPT (AUC = 0.741; 0.771, respectively) and ProCUrE (AUC = 0.746; 0.730, respectively) individually. Further, using c-statistic, we calculated the additive value of ProCUrE to PCPT for diagnosis of PCa using logistic regression, which increased from *c* = 0.741 for PCPT alone to *c* = 0.817 for PCPT with ProCUrE. Similarly, for the detection of CS-PCa (as determined by GS) addition of ProCUrE to PCPT increased from *c* = 0.771 to *c* = 0.822. Both values represent a significant increase (DeLong’s test *p* = 0.011 for diagnostic, *p* = 0.022 for prognostic value) and indicate that the information provided by ProCUrE could further improve current PCPT parameters for prognosticating PCa patients prior to biopsy.

## Discussion

Our study developed a urinary DNA methylation biomarker-based actionable assay, ProCUrE, to identify CS-PCa that would warrant treatment. ProCUrE significantly improves risk stratification with a higher PPV compared to age-adjusted PSA. Patients who are positive for ProCUrE will be more likely to harbor aggressive tumors and thus ProCUrE has the potential to supplement PSA or other tests that focus on NPV. Importantly, ProCUrE has additive value to PSA assessment and to PCPT risk calculator for the detection of aggressive (GS ≥ 7) cancers.

PSA testing cannot reliably distinguish patients that have CS-PCa disease from those that do not require treatment. Therefore, invasive confirmation biopsy is necessary for PCa diagnosis and prognostication. A non-invasive adjunct test to PSA, such as ProCUrE, that can identify patients with CS-PCa would reduce overtreatment and prevent morbidity associated with unnecessary biopsies.

ProCUrE is comprised of the promoter methylation of *HOXD3* and *GSTP1* genes. *HOXD3* is a member of the homeobox gene family of transcription factors which play important roles in morphogenesis and cell adhesion [17, 18], while *GSTP1* is a member of the GST family of metabolic enzymes which function in regulation of cell cycle, DNA repair, and apoptosis [19]. Increased methylation levels of *HOXD3* and *GSTP1* are observed in prostate tumors and are correlated with aggressive PCa and/or adverse clinical outcomes [9, 18, 20, 21]. *GSTP1* methylation has been previously investigated in urine sediments and was found to be PCa specific when compared to benign patients [22].

In a recent study of urinary methylation biomarkers, *APC* and *GSTP1* methylation in conjunction with clinical variables demonstrated 100% NPV for distinguishing GS ≥ 7 PCa [23]. Although this study demonstrated that urine-based DNA methylation markers could be used to prognosticate PCa aggressiveness, their results showed a high (26%) false positive rate compared to only 13.8% false positive rate observed for ProCUrE. Thus, their combined panel of *APC* and *GSTP1* is less than favorable to address the current challenges for managing PCa, specifically, to minimize overtreatment of low-risk patients. Currently available non-invasive tests for PCa diagnosis and prognosis include the Prostate Health Index [24], SelectMDx [25], mpMRI [26], and PCA3. Similar to ProCUrE, SelectMDx is a post-DRE urine-based, two-gene (*HOXC6* and *DLX1*) expression assay that can detect CS-PCa (GS > 6) (AUC = 0.77). The Prostate Health Index (PHI) [27] is a FDA-approved blood test that measures total, free and -2proPSA with greater specificity than free and total PSA for CS-PCa [28]. MpMRI has a high NPV (95%) for GS ≥ 7 tumors [26]. However, high cost and limited availability remain a limitation for implementing mpMRI as a screening tool. The Progensa PCA3 test is the only FDA approved urine-based test for PCa diagnosis. With its high NPV for PCa (90%) [29], PCA3 can prevent unnecessary repeat biopsies. The Mi-Prostate score combines PCA3 and TMPRSS2:ERG fusion with the multivariable Prostate Cancer Prevention Trial risk calculator (PCPT) for prediction of PCa (AUC = 0.762) and high-risk PCa (AUC = 0.779) which is comparable to our ProCUrE assay in the training cohort (AUC = 0.795 for benign vs high-risk PCa, Fig. 2) [30] Additionally, we demonstrated that ProCUrE, when combined with PCPT, has even greater AUCs for diagnosis (0.817) and prognostication (0.822) of PCa than the Mi-Prostate score. However, it should be noted that this comparison was calculated on a subset of the total number of patients that had DRE, family history, and ethnicity information available.

All of the aforementioned tests are promising for PCa diagnosis or prognostication. However, all of these tests focus on NPV for PCa or high-risk PCa. Patients who are above the selected thresholds for these tests remain uncertain with respect to their disease status. Working in conjunction with the above tests or PSA, our ProCUrE assay fulfills a niche by focusing on PPV to offer a distinct advantage in identifying PCa patients with clinically significant tumors. Thus, patients who cannot be ruled out as having indolent tumors could be tested with ProCUrE to assess whether they have aggressive disease. ProCUrE could also be combined with tests such as SelectMDx to build a more comprehensive multivariable urine test in the future. This will improve current clinical PCa patient management once validated in independent studies.

Our study has certain limitations, including the fact that patient cohorts recruited for our study had differences in size and composition (e.g., UEA cohort had patients with higher PSA, GS, T stage compared to other cohorts) and as such, they could not be analyzed as three independent cohorts, despite using consensus recruitment criteria. Histopathological-based cancer diagnosis of biopsies was performed at three different participating centers which may have contributed to some variation in Gleason grading between cohorts. However, our strategy of combining patients from all three cohorts and subsequently randomizing into training and validation cohorts, overcomes these caveats as it ensured that the training and validation sets would include patients representing a broad spectrum of prostate status, from benign with low PSA to very high risk PCa.

Other potential caveats are that benign patients with abnormalities such as high PSA may have contributed to the lack of significance for age-adjusted PSA with PCa diagnosis. Patients who are false positive for ProCUrE +ve status may actually harbor occult tumors. In this regard, follow-up data collection is ongoing for future biopsies and/or MRIs, which will enable assessment of ProCUrE for prediction and confirmation of CS-PCa. Additionally, clinical stage information for the UEA and Dublin cohorts did not differentiate T2a, T2b, and T2c tumors. Therefore, D’Amico criteria was calculated with all T2 patients assigned as intermediate risk. This may have contributed to the lack of significance for ProCUrE to stratify patients based on D’Amico criteria. In previously published studies, we described a 4-gene methylation Classifier Panel (*APC*, *GSTP1*, *CRIP3*, *HOXD8*) in PCa patients monitored by active surveillance (AS) for the prediction of risk-reclassification [12, 31]. We were unable to screen two genes (*CRIP3* and *HOXD8)* from this four-gene classifier panel in the current study due to limitations of DNA samples availability. Similar to our findings in the AS PCa patient cohort, we found that methylation frequencies of *APC* and *GSTP1* in urinary sediment were lower compared to those reported in tissue samples. Lastly, it is difficult to assess the additive value of ProCUrE to PSA for identifying patients based on the CAPRA score and D’Amico criteria, since both nomograms are calculated using PSA leading to strong association with PSA with these risk groups. Both CAPRA and D’Amico criteria are limited in that they require prostate biopsy to calculate risk. ProCUrE is advantageous in this regard since risk assessment can be performed prior to biopsy.

## Conclusion

A non-invasive urine-based assay that can distinguish PCa patients with aggressive, clinically significant disease from those with benign and/or low risk disease would be valuable in reducing morbidity associated with over-diagnosis and preventing under-diagnosis of patients that would benefit from definitive treatment. Our ProCUrE assay could be used to supplement PSA screening and monitoring so those with aggressive disease would be identified early and those without will avoid unnecessary treatment.

## Materials and methods

### Patient cohorts

Participants were prospectively recruited between April 2012 and September 2015, from the University of East Anglia/Norfolk and Norwich University Hospital, UK (UEA cohort, *n* = 194), the University Health Network, Canada (UHN cohort, *n* = 155), and Trinity College, Ireland (Dublin cohort, *n* = 59), together as part of the Movember GAP1 Multi-Center Urine Biomarker (MoGAP-MUB) cohort. Patients underwent prostate TRUS biopsy due to increased PSA and/or abnormal DRE (PSA follow-up time 0–122 months). Benign patients with normal age-adjusted PSA were recruited due to symptoms of BPH or had microhematuria detectable on dipstick only (i.e., not gross hematuria). Less than 10% (39/408) patients had prior biopsies, all other patients were recruited at initial biopsy. Post-DRE first catch urine samples were either collected prior to biopsy or at least 1-month post-biopsy. Samples were mostly collected within 12 months from the date of biopsy. There were two patients that had > 12-month difference between biopsy and sample collection (range 14–146 months) and three patients with unknown biopsy dates. The patient with sample collected 146 months post-biopsy and all patients with unknown biopsy dates were benign patients. Informed consent was obtained following protocols approved by the research ethics boards of all centers and Sinai Health System, Toronto, Canada.

The cut-off for normal PSA (referred to as age-adjusted PSA) was determined following British Association of Urological Surgeon guidelines [15, 16]. Patients were classified based on the following criteria:

Benign indicates patients with negative biopsy.

GS, D’Amico criteria, and CAPRA score were utilized to stratify risk in PCa patients:

GS: low risk (GS ≤ 6), intermediate risk (GS7), and high risk (GS ≥ 8).

D’Amico criteria: low risk (GS ≤ 6 and T1–T2a and PSA < 10 ng/mL), intermediate risk (GS7 or T2b or PSA 10–20 ng/mL), and high risk (GS ≥ or > or PSA > 20 ng/mL) [3].

The CAPRA score is defined as the sum of the following variables: age at diagnosis (< 50 = 0, ≥ 50 = 1), PSA at diagnosis(ng/mL) (≤ 6 = 0, 6.1–10 = 1, 10.1–20 = 2, 20.1–30 = 3, > 30 = 4), biopsy Gleason pattern (no pattern ≥ 4 = 0, secondary pattern ≥ 4 = 1, primary pattern ≥ 4 = 3), clinical T stage (T1 or T2 = 0, ≥ T3 = 1; %core: < 34% = 0, ≥ 34% = 1) [4]. CAPRA risk categories are as follows: low risk (0–2 points), intermediate risk [3–5], and high risk (≥ 6).

Calculation of PCPT risk score

PCPT risk was calculated using the Cleveland Clinic Risk Calculator Library – PCPT Risk Calculator v2.0 [5].

### Urine collection/processing

Up to 50 mL of first catch urine was collected from each patient following DRE and centrifuged at 1200×*g* for 5 min. Urine sediments were separated from supernatant and resuspended in 1 ml of PBS and stored at − 80 °C. Urinary sediment DNA was extracted using the AllPrep DNA/RNA mini-kit (Qiagen Inc.) Bisulfite conversion was as previously described [12].

### MethyLight analysis

Multiplex MethyLight, a methylation-specific qPCR assay was used to determine the methylation levels of *APC*, *GSTP1*, *HOXD3*, *KLK10*, *TBX15*, and *TGFβ2* [32]. *ALU-C4* (*ALU*) was used as a methylation-independent, sodium bisulfite conversion-dependent internal input DNA control.

Primer/probe concentrations, cycling parameters, and data acquisition/analysis were as previously described, using Applied Biosystems 7500 (Life Technologies) [12].

Gene methylation was scored as percent methylated of reference (PMR) according to Eads et al. [33] CpGenome Universal Methylated DNA (EMD Millipore) was used as the positive control and to generate standard curves. Quality control criteria included genes of interest (GOIs) standard curve *R*^2^ > 0.95, ALU *R*^2^ > 0.99, and slope range from − 3.28 to − 4.86. Any sample with a higher cycling threshold (lower quantity) for *ALU* than the least concentrated standard curve point for which all GOI amplified was excluded from analysis. Samples were analyzed in duplicate and were reanalyzed if replicates had a difference in PMR of > 10%. Data development and analysis were carried out in accordance with the Minimum Information for Publication of Quantitative real-time PCR Experiments (MIQE) guideline [34].

### Calculation for ProCUrE

Least absolute shrinkage and selection operator (LASSO) was applied to construct gene models using benign vs CAPRA high-risk (CAPRA-HR) patients in the training cohort. LASSO was used to eliminate genes that had insufficient contribution to the model. The remaining genes (*APC*, *GSTP1*, *HOXD3*, *KLK10*, *TGFβ2*) with non-zero coefficients as determined by LASSO were tested for every possible combination using the generalized linear model in the training cohort to determine their AUC, Akaike information criterion (AIC), and Bayesian information criterion (BIC). An optimal two-gene model consisting of *HOXD3* and *GSTP1*, which had the highest AUC with the lowest AIC and BIC, was selected for further analysis. We developed Prostate Cancer Urinary Epigenetic (ProCUrE) assay, based on the formula:

$$ \mathrm{Intercept}+\mathrm{Coefficient}\left(\mathrm{GSTP}1\right)\ast \mathrm{PMR}\left(\mathrm{GSTP}1\right)+\mathrm{Coefficient}\left(\mathrm{HOXD}3\right)\ast \mathrm{PMR}\left(\mathrm{HOXD}3\right) $$

where the intercept is − 0.8395549, the coefficient for *HOXD3* is 0.1397128, and the coefficient for *GSTP1* is 0.8632709.

Additional comparisons (benign vs PCa, CI vs CS-PCa as determined by GS, CAPRA and D’Amico) were performed in the training cohort (Additional file 2: Figure S2). However, none of these comparisons yielded a model with as robust discriminative value (higher AUC) as observed with benign vs CAPRA-HR comparison. Therefore, we opted for the model constructed using benign vs CAPRA-HR for further analysis.

### Statistical analysis

Spearman’s ρ rank was used to compare PMR, age, %core, prostate volume (cc), and PSA at diagnosis. ROC curve analysis was used to determine ProCUrE’s sensitivity and specificity at every cut-off value. The value with the highest sum of sensitivity and specificity was chosen as the optimized threshold. The same numerical values derived from the training cohort were used in the validation cohort: threshold derived from ROC analysis (threshold value = 0.574264899821094) and intercept and coefficients derived from generalized linear modeling. *χ*^2^ tests were used to determine any significant association with overall cancer status or CS-PCa (≥ intermediate-risk cancer as determined by GS, CAPRA, or D’Amico criteria).

Univariable and multivariable logistic regression was performed to estimate odds ratios and corresponding 95% confidence intervals to assess the ability of individual markers, ProCUrE, and clinical variables to identify CS-PCa patients using the lrm function of the “rms” R package (5.1–2). C-statistic was calculated using ROC curves [35]. DeLong’s test [36] was used to compare significance for c-statistic as part of the roc.test function of the pROC R package (v1.13.0).

LASSO analysis was carried out using the “glmnet” function of the “glmnet” R package (v2.0-13) [37] to determine the optimal value of the penalty coefficient lambda, with 10-fold cross-validation performed using the “cv.glmnet” function. Optimal lambda was chosen as the cross-validated lambda at the minimum binomial deviance. Model assessment was performed using the “ROCR” R package, AUC was determined via bootstrapping with 1000 iterations.

For all described methods, two-sided *p* values of < 0.05 were considered significant. All tests were conducted with IBM SPSS software (SPSS Inc. Released 2014. PASW Statistics for Windows, Version 22.0) or R version 3.4.0 [38]. Reporting recommendations for tumor marker prognostic studies (REMARK) guidelines were followed in analysis [39].

## Additional files

Additional file 1: **Table S1.** Correlations. Spearman’s rank correlations for the PMR values of each biomarker, ProCUrE, and clinical variables Spearman’s ρ *p < 0.05; **p < 0.01. **Table S2.** Average PMR values of individual gene methylation for benign and PCa patients. All genes except *TBX15* was able to significantly differentiate between benign and PCa (Mann Whitney *U p* < 0.05). **Table S3.** Diagnosis (A) and prognostication (B-D) of PCa in the training cohort. (DOCX 27 kb)

Additional file 2: **Figure S1.** Diagnostic and prognostic ability of ProCUrE and age-adjusted PSA in the training cohort. **Figure S2.** Receiver operating characteristic curve analysis of training cohort for (A) benign vs PCa, clinically insignificant vs clinically significant PCa as determined by (B) GS, (C) CAPRA, and (D) D’Amico. (DOCX 291 kb)

## Abbreviations

%core: Percent of cores positive in biopsy
AIC: Akaike information criterion
AS: Active surveillance
AUC: Area under curve
BIC: Bayesian information criterion
CAPRA: Cancer of the Prostate Risk Assessment
CI-PCa: Clinically insignificant prostate cancer
CS-PCa: Clinically significant prostate cancer
DRE: Digital rectal exam
GAP: Global Action Plan
GOI: Gene of interest
GS: Gleason score
IQR OR: Interquartile range odds ratios
LASSO: Least absolute shrinkage and selection operator
MIQE: Minimum Information for Publication of Quantitative real-time PCR Experiments
MoGAP-MUB: Movember GAP1 Multi-Center Urine Biomarker
MpMRI: Multiparametric magnetic resonance imaging
NPV: Negative predictive value
PCa: Prostate cancer
PCPT: Prostate Cancer Prevention Trial
PMR: Percent methylated of reference
PPV: Positive predictive value
ProCUrE: Prostate Cancer Urinary Epigenetic
PSA: Prostate specific antigen
ROC: Receiver operating characteristic
USPSTF: U.S. Preventive Services Task Force
