# Supplementary material for: A urine-based DNA methylation assay, ProCUrE, to identify clinically significant prostate cancer
Source: Clin Epigenetics. 2018 Nov 23;10:147. doi: 10.1186/s13148-018-0575-z (PMC6260648; doi:10.1186/s13148-018-0575-z)
Supplement: Supplementary file 1 — Table S1. Correlations. Spearman’s rank correlations for the PMR values of each biomarker, ProCUrE, and clinical variables Spearman’s ρ *p < 0.05; **p < 0.01. Table S2. Average PMR values of individual gene methylation for benign and PCa patients. All genes except TBX15 was able to significantly differentiate between benign and PCa (Mann Whitney U p < 0.05). Table S3. Diagnosis (A) and prognostication (B-D) of PCa in the training cohort. (DOCX 27 kb) [file 13148_2018_575_MOESM1_ESM.docx]

Table S1 Correlations

Spearman's rank correlations for the PMR values of each biomarker, ProCUrE, and clinical variables Spearman's ρ **p*<0.05; **p<0.01

| Correlations | | | | | | | | | | | | | |
| --- | --- | --- | --- | --- | --- | --- | --- | --- | --- | --- | --- | --- | --- |
|  | | | | | | | | | | | | | |
|  | | | HOXD3 PMR | TGFb2 PMR | GSTP1 PMR | KLK10 PMR | TBX15 PMR | ProCUrE | Age | PSA | %Core | Prostate volume |  |
| Spearman's ρ | APC PMR | Correlation Coefficient | .485** | .724** | .454** | .463** | .427** | .563** | .165** | .034 | .298** | .021 |  |
|  |  | N | 405 | 407 | 407 | 400 | 406 | 405 | 407 | 407 | 212 | 238 |  |
|  | HOXD3 PMR | Correlation Coefficient |  | .458** | .334** | .245** | .336** | .928** | .053 | .062 | .015 | -.049 |  |
|  |  | N |  | 405 | 405 | 399 | 404 | 405 | 405 | 405 | 210 | 236 |  |
|  | TGFb2 PMR | Correlation Coefficient |  |  | .390** | .375** | .377** | .504** | .174** | .052 | .194** | -.015 |  |
|  |  | N |  |  | 408 | 401 | 407 | 405 | 408 | 408 | 212 | 239 |  |
|  | GSTP1 PMR | Correlation Coefficient |  |  |  | .644** | .437** | .583** | .256** | .247** | .411** | .100 |  |
|  |  | N |  |  |  | 401 | 407 | 405 | 408 | 408 | 212 | 239 |  |
|  | KLK10 PMR | Correlation Coefficient |  |  |  |  | .397** | .424** | .151** | .209** | .323** | .027 |  |
|  |  | N |  |  |  |  | 400 | 399 | 401 | 401 | 206 | 233 |  |
|  | TBX15 PMR | Correlation Coefficient |  |  |  |  |  | .405** | .199** | .182** | .335** | .060 |  |
|  |  | N |  |  |  |  |  | 404 | 407 | 407 | 212 | 238 |  |
|  | ProCUrE | Correlation Coefficient |  |  |  |  |  |  | .130** | .137** | .184** | -.035 |  |
|  |  | N |  |  |  |  |  |  | 405 | 405 | 210 | 236 |  |
|  | Age | Correlation Coefficient |  |  |  |  |  |  |  | .325** | .283** | .364** |  |
|  |  | N |  |  |  |  |  |  |  | 408 | 212 | 239 |  |
|  | PSA | Correlation Coefficient |  |  |  |  |  |  |  |  | .436** | .299** |  |
|  |  | N |  |  |  |  |  |  |  |  | 212 | 239 |  |
|  | %Core | Correlation Coefficient |  |  |  |  |  |  |  |  |  | -.058 |  |
|  |  | N |  |  |  |  |  |  |  |  |  | 140 |  |

Table S2. Average PMR values of individual gene methylation for Benign and PCa patients. All genes except *TBX15* was able to significantly differentiate between Benign and PCa (Mann Whitney U *p*<0.05)

|  | APC PMR | HOXD3 PMR | TGFb2 PMR | GSTP1 PMR | KLK10 PMR | TBX15 PMR |
| --- | --- | --- | --- | --- | --- | --- |
| Benign | 0.24 | 1.73 | 0.22 | 0.11 | 0.13 | 0.22 |
| PCa | 2.47 | 7.04 | 1.07 | 5.53 | 3.60 | 3.20 |
| Mann Whitney U P-value | <0.001 | <0.001 | <0.001 | <0.001 | 0.011 | 0.283 |

Table S3 Diagnosis (A) and prognostication (B-D) of PCa in the training cohort

Positive (PPV) and Negative (NPV) predictive values for ProCUrE and Age adjusted PSA in the training cohort separating Benign vs PCa (A); clinically insignificant (Benign and low-risk) vs clinically significant (intermediate- and high-risk) and low-risk vs clinically significant (intermediate- and high-risk) as determined by GS, CAPRA score, D'Amico criteria. (χ^2^ p-values for these comparisons could be found in Supplemental Fig. 1)

| A | PPV | NPV |
| --- | --- | --- |
| Benign vs PCa | | |
| ProCUrE | 90.4% | 54.4% |
| Age adjusted PSA | 60.4% | 61.8% |
| B | | |
| GS Clinically insignificant vs Clinically significant | | |
| ProCUrE | 63.5% | 75.8% |
| Age adjusted PSA | 37.5% | 82.9% |
| GS6 vs GS≥7 | | |
| ProCUrE | 70.2% | 46.9% |
| Age adjusted PSA | 62.1% | 55.2% |
| Benign, GS6, GS7(3+4) vs GS≥7 (4+3) | | |
| ProCUrE | 57.4% | 76.5% |
| Age adjusted PSA | 39.7% | 86.2% |
| C | | |
| CAPRA Clinically insignificant vs Clinically significant | | |
| ProCUrE | 59.40% | 73.60% |
| Age adjusted PSA | 42.20% | 86.80% |
| CAPRA low-risk vs Intermediate- and high-risk | | |
| ProCUrE | 83.0% | 33.7% |
| Age adjusted PSA | 78.4% | 55.2% |
| D | | |
| D'Amico Clinically insignificant vs Clinically significant | | |
| ProCUrE | 75.0% | 69.8% |
| Age adjusted PSA | 47.4% | 82.9% |
| D'Amico low-risk vs Intermediate- and high-risk | | |
| ProCUrE | 76.00% | 38.90% |
| Age adjusted PSA | 72.10% | 55.00% |
