# Supplementary material for: A urine-based DNA methylation assay, ProCUrE, to identify clinically significant prostate cancer
Source: Clin Epigenetics. 2018 Nov 23;10:147. doi: 10.1186/s13148-018-0575-z (PMC6260648; doi:10.1186/s13148-018-0575-z)
Supplement: Supplementary file 2 — Figure S1. Diagnostic and prognostic ability of ProCUrE and age-adjusted PSA in the training cohort. Figure S2. Receiver operating characteristic curve analysis of training cohort for (A) benign vs PCa, clinically insignificant vs clinically significant PCa as determined by (B) GS, (C) CAPRA, and (D) D’Amico. (DOCX 291 kb) [file 13148_2018_575_MOESM2_ESM.docx]

Figure S1 Diagnostic and prognostic ability of ProCUrE and age adjusted PSA in the training cohort.

1. The percent false- and true-positive for ProCUrE or age adjusted PSA separating benign and PCa patients.
2. The percent of patients positive for ProCUrE or age adjusted PSA for clinically insignificant (benign and low-risk) vs clinically significant (intermediate- and high-risk) based on Gleason score and GS6 vs GS≥7; benign-GS7(3+4) vs GS≥7(4+3).
3. & (D) The percent of patients positive for ProCUrE or age adjusted PSA for clinically insignificant (benign and low-risk) vs clinically significant (intermediate- and high-risk) and low-risk vs intermediate- and high-risk as determined by CAPRA score (C), and D’Amico (D). N=267, χ^2^**p*<0.05, ***p*<0.01, ****p*<0.001

A)


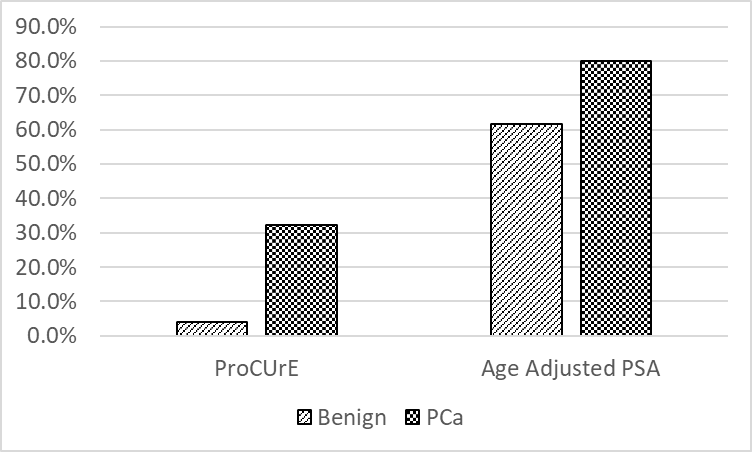


**

***

B)


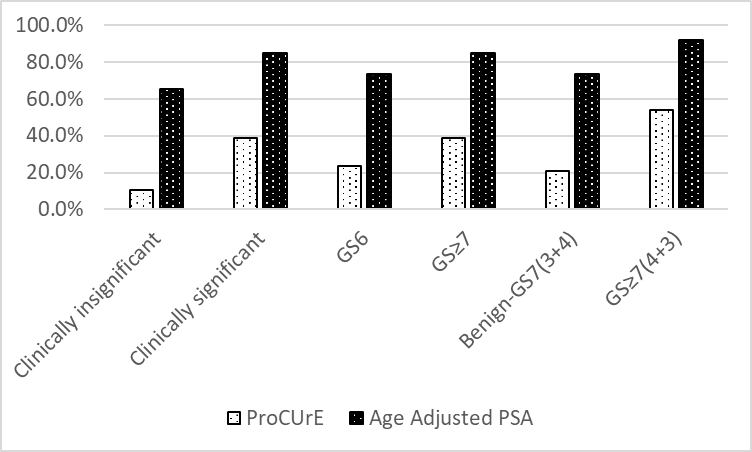


***

**

*

***

**

C)


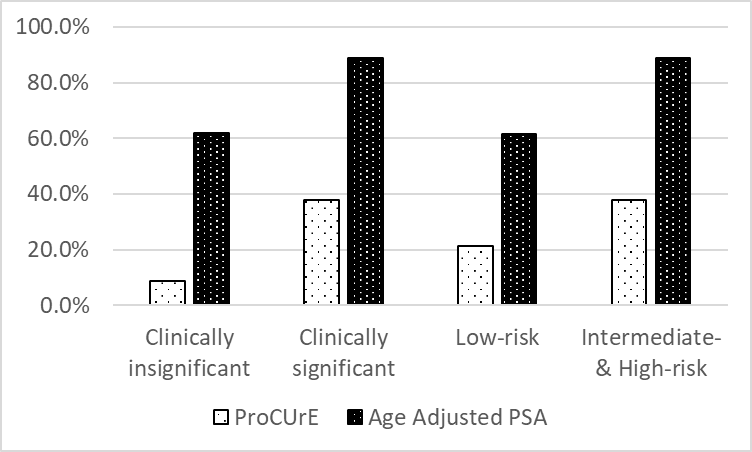


***

***

***

*

D)


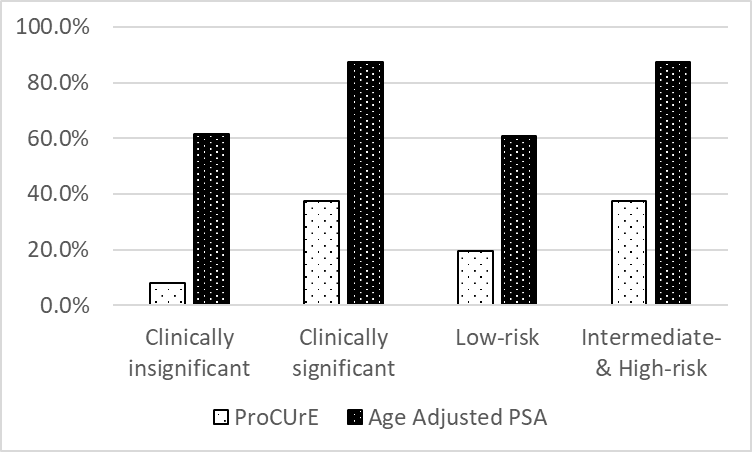


***

***

***

*

Figure S2 Receiver Operating Characteristic curve analysis of training cohort for (A) benign vs PCa, Clinically insignificant vs Clinically significant PCa as determined by (B) GS, (C) CAPRA, and (D) D’Amico.

A)


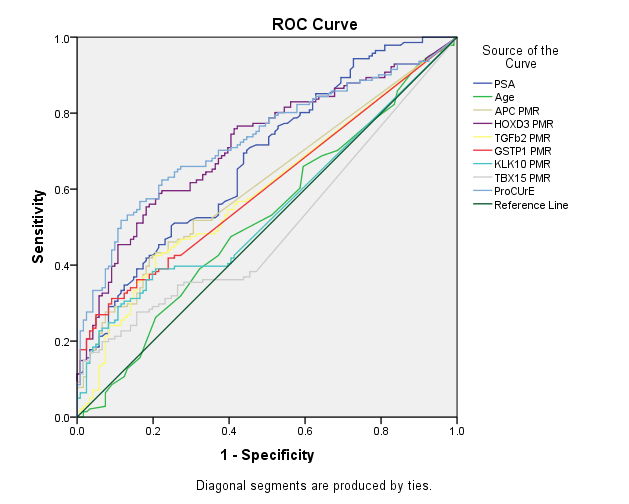


| Benign vs PCa | AUC | 95% C.I. | | p-value |
| --- | --- | --- | --- | --- |
| PSA | 0.679 | 0.615 | 0.743 | 5.85E-07 |
| Age | 0.523 | 0.453 | 0.594 | 5.13E-01 |
| APC PMR | 0.617 | 0.549 | 0.684 | 1.11E-03 |
| HOXD3 PMR | 0.718 | 0.656 | 0.779 | 1.23E-09 |
| TGFb2 PMR | 0.596 | 0.527 | 0.664 | 7.52E-03 |
| GSTP1 PMR | 0.605 | 0.538 | 0.673 | 3.30E-03 |
| KLK10 PMR | 0.552 | 0.482 | 0.621 | 1.51E-01 |
| TBX15 PMR | 0.502 | 0.431 | 0.572 | 9.63E-01 |
| ProCUrE | 0.730 | 0.669 | 0.791 | 1.37E-10 |

B)


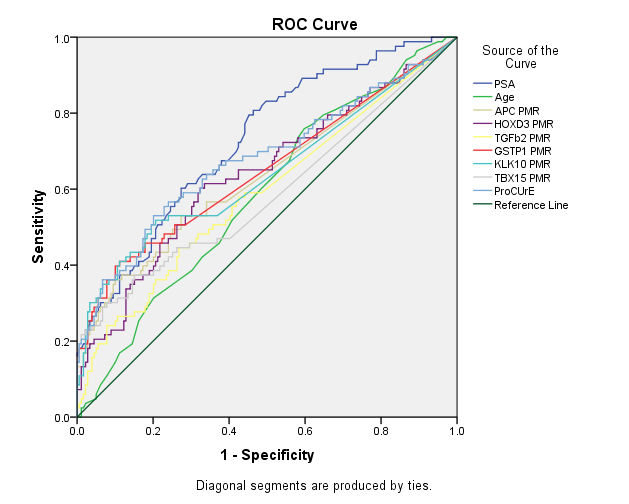


| CI-PCa vs CS-PCa (GS) | AUC | 95% C.I. | | p-value |
| --- | --- | --- | --- | --- |
| PSA | 0.727 | 0.663 | 0.792 | 3.28E-09 |
| Age | 0.586 | 0.513 | 0.658 | 2.60E-02 |
| APC PMR | 0.637 | 0.560 | 0.714 | 3.71E-04 |
| HOXD3 PMR | 0.640 | 0.565 | 0.715 | 2.71E-04 |
| TGFb2 PMR | 0.590 | 0.513 | 0.667 | 1.93E-02 |
| GSTP1 PMR | 0.649 | 0.572 | 0.726 | 1.00E-04 |
| KLK10 PMR | 0.643 | 0.564 | 0.721 | 2.02E-04 |
| TBX15 PMR | 0.588 | 0.508 | 0.668 | 2.17E-02 |
| ProCUrE | 0.678 | 0.602 | 0.753 | 3.74E-06 |

C)


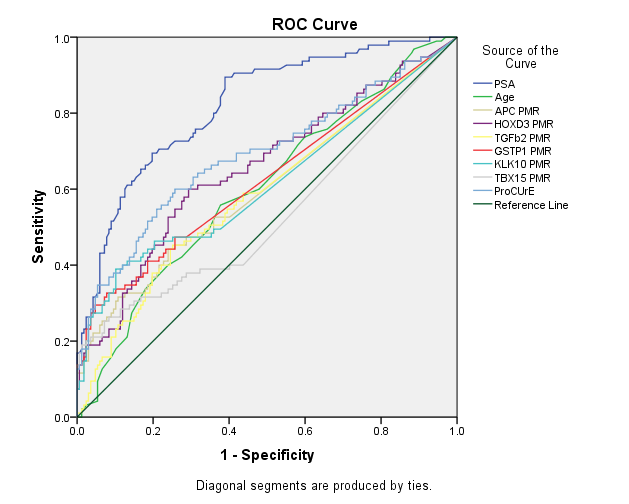


| CI-PCa vs CS-PCa (CAPRA) | AUC | 95% C.I. | | p-value |
| --- | --- | --- | --- | --- |
| PSA | 0.820 | 0.768 | 0.872 | 7.31E-18 |
| Age | 0.603 | 0.532 | 0.674 | 5.73E-03 |
| APC PMR | 0.604 | 0.529 | 0.678 | 5.29E-03 |
| HOXD3 PMR | 0.656 | 0.585 | 0.727 | 2.74E-05 |
| TGFb2 PMR | 0.590 | 0.517 | 0.664 | 1.52E-02 |
| GSTP1 PMR | 0.626 | 0.552 | 0.700 | 7.23E-04 |
| KLK10 PMR | 0.614 | 0.539 | 0.690 | 2.09E-03 |
| TBX15 PMR | 0.533 | 0.456 | 0.610 | 3.76E-01 |
| ProCUrE | 0.689 | 0.618 | 0.760 | 3.77E-07 |

D)


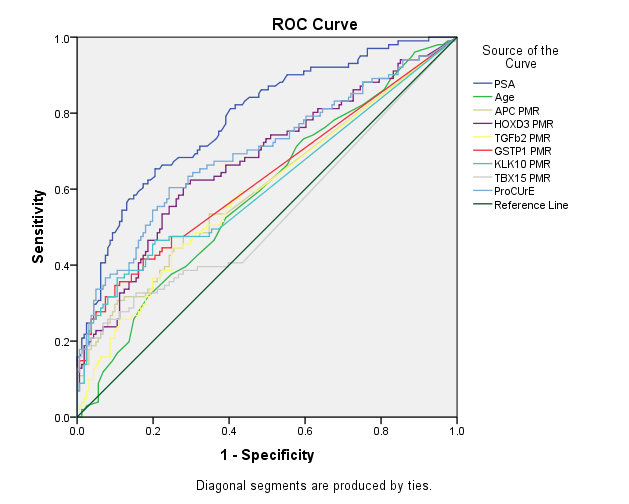


| CI-PCa vs CS-PCa (D'Amico) | AUC | 95% C.I. | | p-value |
| --- | --- | --- | --- | --- |
| PSA | 0.786 | 0.729 | 0.843 | 6.61E-15 |
| Age | 0.587 | 0.517 | 0.658 | 1.73E-02 |
| APC PMR | 0.606 | 0.534 | 0.679 | 3.77E-03 |
| HOXD3 PMR | 0.674 | 0.606 | 0.743 | 2.08E-06 |
| TGFb2 PMR | 0.594 | 0.522 | 0.666 | 1.04E-02 |
| GSTP1 PMR | 0.630 | 0.557 | 0.702 | 4.15E-04 |
| KLK10 PMR | 0.613 | 0.539 | 0.686 | 2.17E-03 |
| TBX15 PMR | 0.538 | 0.462 | 0.613 | 3.05E-01 |
| ProCUrE | 0.697 | 0.628 | 0.765 | 8.42E-08 |
